# Photodynamic Therapy for Basal Cell Carcinoma in Recessive Dystrophic Epidermolysis Bullosa

**DOI:** 10.5402/2011/346754

**Published:** 2011-04-27

**Authors:** Myn Wee Lee, George Varigos, Peter Foley, Gayle Ross

**Affiliations:** ^1^Department of Dermatology, The Royal Melbourne Hospital, Grattan Street, Parkville, VIC 3050, Australia; ^2^Department of Medicine, The University of Melbourne, Parkville, VIC 3050, Australia; ^3^Department of Dermatology, St. Vincent's Hospital, Fitzroy, VIC 3065, Australia; ^4^Skin and Cancer Foundation, 80 Drummond Street, Carlton, VIC 3053, Australia

## Abstract

A 22-year-old male with recessive dystrophic epidermolysis bullosa with a large superficial and nodular basal cell carcinoma on his right forehead was treated with photodynamic therapy. The treatment was well tolerated, and the site healed well. Patients with epidermolysis bullosa are at increased risk of developing skin cancers, particularly squamous cell carcinomas. However, basal cell carcinomas are rare in recessive dystrophic epidermolysis bullosa. As patients with epidermolysis bullosa have recurrent blistering and poor wound healing, surgery may not be the optimal choice in treating skin cancers. We present this case to highlight that photodynamic therapy may be a helpful and safe technique in the treatment of superficial skin cancers in patients with epidermolysis bullosa, as an alternative to more radical methods.

## 1. Introduction

Epidermolysis bullosa (EB) encompasses a group of inherited skin fragility disorders, characterised by blistering of the skin and mucous membranes following minimal trauma [1]. EB is divided into distinct subtypes based on the level of blister formation within the skin, each subtype having different genetic mutations. Dystrophic epidermolysis bullosa (DEB) is caused by mutations in the gene COL7A1, which encodes type VII collagen, the predominant anchoring fibril protein at the dermal-epidermal junction [2]. Therefore, cleavage occurs beneath the lamina densa of the cutaneous basement membrane. Abnormality in the number and appearance of anchoring fibrils which serve as adhesion structures leads to extreme mucocutaneous fragility and formation of chronic wounds, which have an impaired ability to heal [3]. Blisters within the sublamina densa region of the skin elicit a fibrosing response in the dermis, which explains the tendency of healing with extensive scarring [2]. DEB is inherited in autosomal dominant or recessive pattern, which contributes to the variable clinical spectrum of the disease.

## 2. Case Report

Our patient was diagnosed with Hallopeau-Siemens dystrophic recessive epidermolysis bullosa (DREB) in infancy, characterized by severe fragility and blistering of his skin. Complications have included mitten deformities of the hands and contractures in his hips, knees, and ankles, resulting in him being wheelchair bound for more than 10 years. He has recurrent oesophageal strictures, anaemia (chronic disease and iron deficiency), and secondary hypogonadism. Given his medical condition, the patient's lifestyle is predominantly indoors, with minimal sun exposure. He has no known family history of skin cancers and has never been exposed to gamma radiation in the past.

**Figure 1:**
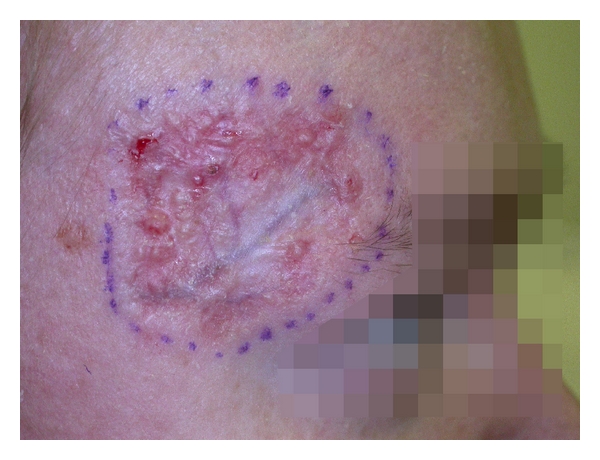
Superficial basal cell carcinoma on patient's right forehead, with nodular growth pattern at the periphery.

A chronic lesion was noted on routine skin check on the patient's right forehead (Figure 1). The patient had been applying a potent topical steroid to the area. Biopsies taken from the centre of the lesion confirmed a superficial basal cell carcinoma (BCC), whilst the edge had a nodular growth pattern.

Given the size of the tumour, which would have required excision and grafting, it was hypothesized that photodynamic therapy (PDT) may potentially be an effective treatment with improved healing and better cosmesis than excision.

Photodynamic therapy was performed. The area was marked with a 5 mm margin. No curettage was performed. Methyl-ALA (Metvix) cream was applied to the area and allowed to penetrate for 3 hours under an occlusive dressing. The area was then irradiated using an Aktilite lamp for 8 minutes 45 seconds at 37 J/cm^2^ and the whole procedure was repeated two weeks later. The patient experienced an inflammatory reaction expected with PDT. Following the treatment, the site healed steadily over the following 3-4 weeks, with minimal scarring. The entire procedure was well tolerated by the patient and he did not report any significant side effects.

**Figure 2:**
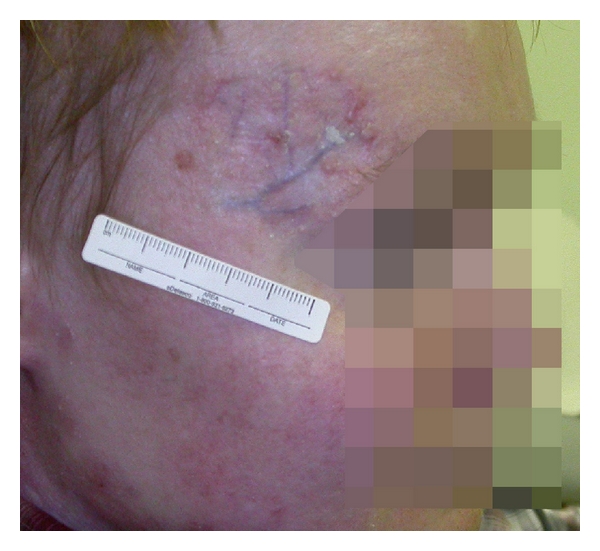
The same lesion 4 months after PDT.

**Figure 3:**
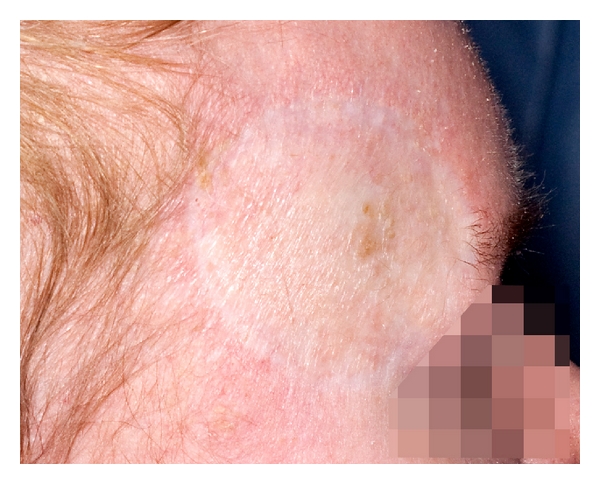
Postexcision and split skin grafting of the residual nodular basal cell carcinoma.

On a subsequent follow-up visit 4 months later, it was noted that there was some residual nodular BCC at the edge of the lesion, which had not responded to the PDT (Figure 2). This was confirmed on biopsy, which revealed granulation tissue in the centre of the lesion, with nodular BCC at the edges. A trial of Imiquimod cream was commenced for a duration of six weeks. The cream was applied five days a week (Monday to Friday), once daily. The patient reported an inflammatory reaction and slight improvement in the appearance of the lesion. Twelve months after the PDT, the patient underwent excision and split skin grafting of the entire area due to continued presence of BCC. Histology confirmed BCC, and the lesion was completely excised. The recipient site healed uneventfully, although the cosmesis was poor (Figure 3). The graft donor site remained an area of poor healing 1 year postoperatively.

## 3. Discussion

In severe generalised recessive DEB (Hallopeau-Siemens), as in our patient, extensive bullae develop from infancy. Clinical presentations include blisters, scarring associated with milia formation, chronic erosions, and ulcers. Repeated blistering and progressive scarring can result in fusion of digits, which may progress to contractures and deformities. Other features include alopecia associated with scarring of the scalp and blistering and stricture of the oesophagus resulting in dysphagia and obstruction.

The most serious complication of DEB is the development of multiple squamous cell carcinomas (SCCs), which is the major cause of morbidity and mortality in these patients [4]. The formation of SCCs is associated with chronic, nonhealing wounds, as present in patients with EB. Therefore it is crucial that malignant lesions are identified early and treated aggressively in patients with EB. However, assessment of skin cancers in EB patients is often difficult, due to widespread ulceration, scarring, and crusting present in these patients [4].

Interestingly, BCCs are uncommon. The National EB Registry in the United States, which has 3280 patients listed, failed to identify any RDEB patients with BCC. Cases of BCC are seen in patients with severe EB simplex [5]. This is possibly explained by the fact that blistering in EB simplex occurs within the basal keratinocytes, from which BCCs are known to arise [5].

Given our patient's young age and absence of significant risk factors, it is unusual that he developed a BCC on his forehead. There are several theories as to why this may have occurred. It is widely accepted that BCCs are ultraviolet (UV) induced; however, there are various genes implicated in the carcinogenesis of BCCs. Mutations of the TP53 tumour suppressor gene and the sonic hedgehog pathway (SHH) genes PTCH and SMOH are found in both sporadic and hereditary forms of BCCs [6]. A study done by Reifenberger et al. found that while 72% of TP53 mutations were presumably UV induced, the “UVsignature” was present in only 40% of PTCH and SMOH mutations [6]. This suggests that there may be other mechanisms, other than UVR that trigger tumourigenesis in BCCs. Genetic analysis was not performed in this case, if carried out could possibly shed some light on this issue.

There have been rare reports of BCCs developing within chronic wounds; however, this is most commonly associated with chronic venous ulcers on the legs [7]. Whether or not this is as in our patient's case is unclear. It seems more likely that the BCC had been present for a significant period of time but had been mistaken for a chronic wound and therefore allowed to proliferate. The potent topical steroid use may also have resulted in the lesion becoming larger and more atrophic than usual.

Topical photodynamic therapy has been approved in 18 countries worldwide for treatment of various skin cancers, including BCC [8]. PDT when employed in treatment of BCCs delivers superior cosmesis than other modes of therapy including surgery and cryotherapy. 87–98% of nBCC had good/excellent cosmesis at 5 years compared with 54% of patients who underwent surgery [8]. However, efficacy of PDT is higher in treating superficial BCCs compared to nodular BCCs, with surgery remaining the gold standard for treatment of nBCC [8]. It is felt that the role of PDT in nBCC is where surgery is suboptimal and cosmesis is important, such as in our patient. Given his medical condition, there was a high likelihood that surgery would result in a wound that was slow to heal.

There has been a lack of the literature addressing the employment of PDT in treatment of skin cancers in patients with EB. Souza and colleagues reported a case of Bowen's disease on the ring finger of a female patient with non-Hallopeau-Siemens recessive DEB, which was treated with 5-aminolaevulinic acid PDT with good clinical outcome. The treated area healed within 4 weeks, with no recurrence of BD at 2-year followup [9]. There are no cases of PDT use in HS-RDEB or for BCCs in patients with EB.

## 4. Conclusion

We believe that this case demonstrates the potential efficacy and healing of a large superficial and nodular BCC in a patient with HS-RDEB. Although surgery unfortunately could not be avoided, we would like to highlight that the reaction generated by PDT was very well tolerated by the patient, and the lesion healed completely. There are several reasons why he ultimately failed to clear the nBCC component. Firstly, the lesion was not curetted due to concerns about skin fragility. This may have hampered control of the nodular component. Secondly, the margin and amount of Metvix may have been insufficient. Perhaps a further PDT treatment may have been successful when combined with curettage. However, as this was a large BCC in a relatively high risk site, it was not felt that further risks could be taken, and excision and grafting was deemed necessary. As such, surgery remains the first-line treatment for nBCC; however, we feel that PDT warrants consideration when there is a role for other methods.

##  Conflict of Interests

Associate Professor Peter Foley has accepted from a sponsor, pharmaceutical company or other organizations the following:

- consultancy fee from the Medical Advisory Board of Galderma Australia and Photocure ASA (Norway), regarding PBS listing of Metvix,
- fee for speaking, conference registration fees and travel/accommodation expenses from the Speakers Bureau for presentations at local, national, and international meetings,
- fee for arranging education, namely, for hosting preceptorship for overseas dermatologists to train in photodynamic therapy.

##  Consent

This case report and all supporting images have been submitted/published with written informed consent from the patient.
